# Heterogeneous pattern of DNA methylation in developmentally important genes correlates with its chromatin conformation

**DOI:** 10.1186/s12867-016-0078-4

**Published:** 2017-01-11

**Authors:** Puja Sinha, Kiran Singh, Manisha Sachan

**Affiliations:** 1 Department of Biotechnology, Motilal Nehru National Institute of Technology, Allahabad, 211004 India; 2 Department of Molecular and Human Genetics, Banaras Hindu University, Varanasi, India

**Keywords:** DNA methylation, Chromatin conformation, Chromatin-immunoprecipitation (ChIP), Sox11, C-mos, HoxB5, Tissue-specific gene expression

## Abstract

**Background:**

DNA methylation is a major epigenetic modification, playing a crucial role in the development and differentiation of higher organisms. DNA methylation is also known to regulate transcription by gene repression. Various developmental genes such as c-mos, HoxB5, Sox11, and Sry show tissue-specific gene expression that was shown to be regulated by promoter DNA methylation. The aim of the present study is to investigate the establishment of chromatin marks (active or repressive) in relation to heterogeneous methylation in the promoter regions of these developmentally important genes.

**Results:**

Chromatin-immunoprecipitation (ChIP) assays were performed to immuno-precipitate chromatin by antibodies against both active (H3K4me3) and repressive (H3K9me3) chromatin regions. The analysis of ChIP results showed that both the percentage input and fold enrichment of activated chromatin was higher in tissues expressing the respective genes as compared to the tissues not expressing the same set of genes. This was true for all the genes selected for the study (c-mos, HoxB5, Sox11, and Sry). These findings illustrate that inconsistent DNA methylation patterns (sporadic, mosaic and heterogeneous) may also influence gene regulation, thereby resulting in the modulation of chromatin conformation.

**Conclusions:**

These findings illustrate that various patterns of DNA methylation (asynchronous, mosaic and heterogeneous) correlates with chromatin modification, resulting in the gene regulation.

## Background

DNA methylation is a major component of epigenetic mechanism which is stably inherited and playing important roles during transcriptional regulation [1, 2]. This event implicates the cytosine residues to be directly modified which is immediately followed by guanine residues (CpGs). These CpG dinucleotides are extremely underrepresented in mammalian genomes and are usually present in small stretches known as CpG islands. These CpG islands are found in approximately 70% of annotated gene promoters and are normally unmethylated in normal somatic tissues. Methylation of these CpG targets leads to loss of gene expression during embryonic development [3]. DNA methylation patterns are well regulated, non-random and tissue-specific which is consistent with its functional importance [4]. DNA methylation is also involved in other fundamental processes like X chromosome inactivation, genomic imprinting, suppression of retrotransposon elements, and is essential for normal development [5]. The principal importance of DNA methylation has been studied in myriad of biological context and the mechanism involves the influence of overall chromatin structure. DNA methylation causes the attraction of repressive complexes and the recruitment of methyl CpG binding proteins (including MeCP2) thereby making the chromatin structure inaccessible for binding of transcription factors leading to the formation of closed chromatin structure [6]. Lucifero et al. [7, 8] reported differences in DNA methylation between maternal and paternal alleles of many imprinted genes (Snrpn, Mest and Peg3). Acquisition of methylation was asynchoronous and heterogeneous at these different genes however the varied patterns of methylation on this subset of imprinted genes have not been analyzed for their role in regulating gene expression. Snrpn methylation data showed unique mosaic pattern of specific cytosine methylation during post natal oocyte development. E-cadherin, a tumor suppressor gene, showed de novo methylation of upstream and downstream regions in neoplastic tissues as predominantly methylated/unmethylated CpG islands [9].

Alternatively DNA methylation plays a pivotal role in embryonic development and differentiation. Developmental genes belonging to different gene families such as Hox and Sox family elicits distinct developmental programs to regulate developmental plasticity during evolution. Various developmentally important genes have been selected for the present study (HoxB5, c-mos, Sox11 and Sry) showing varied temporal and spatial tissue-specific gene expression profile and this variation has been shown to be influenced by DNA methylation patterns directly or indirectly during ontogenesis [11–14].

Therefore the present study focuses to understand the correlation of inconsistency and heterogeneity in DNA methylation patterns during developmental time with altered chromatin conformation and thereby gene regulation. However no reports were found to support this evidence enlightening its relevance in regulation of gene expression and chromatin modifications. Distribution of active (open) or inactive (closed) chromatin marks are studied using ChIP (chromatin-immunoprecipitation) assay for gaining insight towards a gross correlation between the heterogeneous methylation pattern and the distribution of the two chromatin states leading to transcriptional regulation.

## Methods

### Genomic DNA extraction

The Parkes strain of mouse was used in this study. Work was approved by the Institutional Ethics Committee (IEC, Ref No. 176/R&C/13-14) of Motilal Nehru National Institute of Technology Allahabad and the Project Ref. No. is BT/PR13317/GBD/27/252/2009. Mice were randomly bred and maintained under laboratory conditions. Animals were killed by cervical dislocation and different somatic and germinal tissues of both fetal and adult were excised for DNA isolation. Mouse genomic DNA was isolated in three independent sets from adult tissues while 6–7 fetuses were pooled in fetal/neonatal stage especially whole mesonephros gonadal complex from 12.5dpc embryos. DNA was isolated with the help of standard protocol using Proteinase K digestion (50 μg/ml) at 37 °C for 12–14 h. Phenol: chloroform: isoamylalcohol/chloroform: isoamyl alcohol extraction was done at 25 °C. Finally, DNA precipitation was done by adding 1/30th volume of 3 M sodium acetate (pH 5.0) along with two volumes of absolute chilled ethanol.

### Sodium bisulfite treatment

Sodium bisulfite treatment converts cytosine residues to uracil except the methylated cytosine residues which imparts unique distinguishing pattern between methylated and non-methylated cytosines in DNA. The bisulfite conversion was done using the BisulFlash™ DNA Modification Kit (Cat. P-1026, USA) according to manufacturer’s instructions. 400–500 ng of input DNA was used as starting template for bisulfite conversion reaction. The bisulfite converted DNA approx. 1.0–2.0 μl was used for each PCR reaction and was amplified in duplicates which was then pooled together during purification. The primers (forward and reverse each with 20 µM) for c-mos gene were as follows: Region (1970–2189 bp): FP 5′-TGTTTTATGTGATTGTTTTATTTG-3′, RP 5′-CACAAAAACACCATAATAAATAAC-3′. Region (2242 bp-2537 bp): FP 5′-ATTTATTATGGTGTTTTTGTGGTTA-3′, RP 5′-ATTCACTAACTTCAAATCCAAATAC-3′. PCR conditions include denaturation @ 94 °C, annealing @ 58 °C and 54 °C for region I and II respectively, extension @ 72 °C repeated for 30 cycles.

### Cloning and sequencing of positive clones

Amplified PCR products were gel excised and purified from low melting agarose using a DNA purification kit (nucleopore) as per instructions manual. The ligation of purified PCR product was done in T-vector using an InsTAclone™ PCR cloning kit (Thermo Scientific USA) and the ligated product was finally transformed and cloned in *E.coli DH5α*. Positive transformed clones were selected on the basis of blue-white screening and plasmid isolation was done using an alkaline lysis method (Thermo Scientific Plasmid Miniprep Kit). Sequencing of 8-10 independent clones were done by automated DNA sequencer (ABI 3130 genetic analyzer). Plasmid DNA 50–100 ng was used for sequencing with BigDye® Terminator v3.1 Cycle Sequencing Kit as per manufacturer’s instructions.

### RNA isolation and quantitative real time PCR

Total RNA was isolated from mouse tissues using TRI reagent (sigma) according to the manufacturer’s protocol. Concentration of RNA was determined spectrophotometrically at OD 260 nm both before and after DNAse treatment. RNA was reverse transcribed in equal amount using oligo-dT(18) primers and 200 U of MMLV Reverse transcriptase (NEB). cDNA 0.5 µl was used in PCR amplification using SsoFast™ EvaGreen Supermix with Low ROX (2X) along with 5 µM each of forward and reverse primers in real time step one plus PCR machine (ABI). PCR reactions were carried out in triplicates for both *GAPDH* and *c*-*mos*. Expression of mRNA level was estimated and normalized relative to the mRNA level of *GAPDH*. The average of the cycle threshold (Ct) values and standard deviation were determined. Fold change in gene expression was calculated according to 2^− ∆∆Ct^ method [10]. The primer sequences of *GAPDH* are FP: 5′GGAGCCAAACGGGTCATCATCTC3′ and RP-5′GAGGGGCCATCCACAGTCTTCT 3′; *c*-*mos gene* FP 5′-TACGCCACGACAACATAGTTCG-3′ RP 5′-CTTGCTCACTGATCAAAATGTTGG-3′.

### Chromatin-immunoprecipitation (ChIP)

ChIP assay was performed according to the instructions manual (Diagenode ChIP kit Cat. No. kch-orgHIS-012). Chromatin was isolated from different somatic (brain, spleen and kidney) and germinal tissues (testis) of adult, fetal and neonatal stages of mouse. The excised tissues were homogenized and subjected to collagenase treatment (50–200 U/ml) followed by incubation for 2–3 h at 37 °C. Single cell suspension was made by pipetting during the incubation time and cell counting was performed using haemocytometer. The minimum number of cells required to perform ChIP experiments is 1 × 10^6^ cells. Cell cross-linking was done by adding 37% formaldehyde (w/v, final concentration 1%) kept for 10 min at 25 °C on a rotating wheel followed by quenching with 1.25 M glycine (final concentration 125 mM) for 5 min at 25 °C, centrifuged at 4 °C for 5–8 min. Supernatant was discarded and the cell pellet was resuspended in lysis buffer (containing protease inhibitors). The cell suspension was subjected to sonication using a sonicator (SKN-IIDN) at the rate of 3 s ON/1 s OFF for 3–4 cycles for obtaining the desired chromatin range from 200–800 bp. The sheared chromatin was then processed for pre-clearing by adding an IP-incubation mix and pre-blocked beads. Antibodies specific for capturing the desired protein and interacting DNA were used (H3K4me3, Diagenode MAb-152-050 and H3K9me3, Diagenode, MAb-146-050, concentration 1 µg/µl). Negative control IgG antibody (Diagenode, C15400001 (C15200001) was used which binds with non-specific target and the associated DNA fragments were immuno-precipitated. The addition of specific antibodies was followed by incubation on a rotating wheel at 4 °C for overnight. Bead washing with wash buffer—1, 2 and 3 removes non-associated DNA fragments and Protein/DNA complexes were found to get eluted from pre-blocked beads by the addition of elution buffer. The eluted complex was reversibly cross-linked and purified using phenol: chloroform: iso-amyl alcohol/chloroform: iso-amyl alcohol. DNA fragments were precipitated by adding DNA precipitant, DNA co-precipitant and absolute chilled ethanol. The DNA pellet was resuspended in 30 µl of milliQ water and the relative amount of specifically immunoprecipitated DNA was analyzed through PCR amplification using quantitative real-time PCR (ABI step one plus) with 1.0 µl of DNA, SsoFast™ EvaGreen Supermix (2X) with Low ROX (Biorad) and gene specific primers forward and reverse 5 µM each. Control primers *GAPDH* (c17021045, Diagenode used as positive control against activated chromatin regions) and *TSH2β* (c17021042, Diagenode used as positive control against repressed chromatin regions) were used. The percentage input and fold enrichment was calculated which represents the enrichment of certain histone modifications on specific region using the ChIP reactions performed in triplicate.

The primers used for various ChIP reactions in different developmental genes were shown in Table 1.

**Table 1:** Shows the primer sequence of different genes used for ChIP-qPCR reactions

GENE	Forward primer	Reverse primer
c-mos	5′-TGGGGAAGTGCCTCAAGTAT-3′	5′-AGGTCCAAGTGCAAAATGCT-3′
HoxB5	5′-AGTCCCTGCCCTGCACTAA-3′	5′-GCCTCGTCTATTTCGGTGAA-3′
Sox11	5′-TCCAGGTCCTTAT CCACCAG-3′	5′-GACGACCTCATGTTCG ACCT-3′
Sry	5′-GTCAAGCGCCCCATGAAT-3′	5′-CAGCTGCTTGCTGATCTCTG-3′

## Results

We have selected three more developmentally important genes whose methylation pattern has been already studied in different tissues include kidney, brain, spleen, testis and mesonephros gonadal cells (MGCs) [11–13]. In HoxB5 methylation analysis was performed in fetal and adult stages of mouse kidney and spleen tissues whereas the same analysis was carried out in fetal and adult stages of mouse brain and kidney tissues in Sox11. Promoter methylation pattern of Sry was determined in mouse 12.5 dpc mesonephros gonadal complex (MGCs) and in adult testis. Comparing the methylation status of HoxB5 and Sox11, higher methylation was found in adult as compared to fetus. In Sry, less methylation was present in fetal gonads as compared to fetal liver. The work done in the above cited references showed a direct inverse correlation between tissue-specific developmental methylation pattern and gene expression.

### Methylation and expression pattern of c-mos gene

**Fig. 1:**
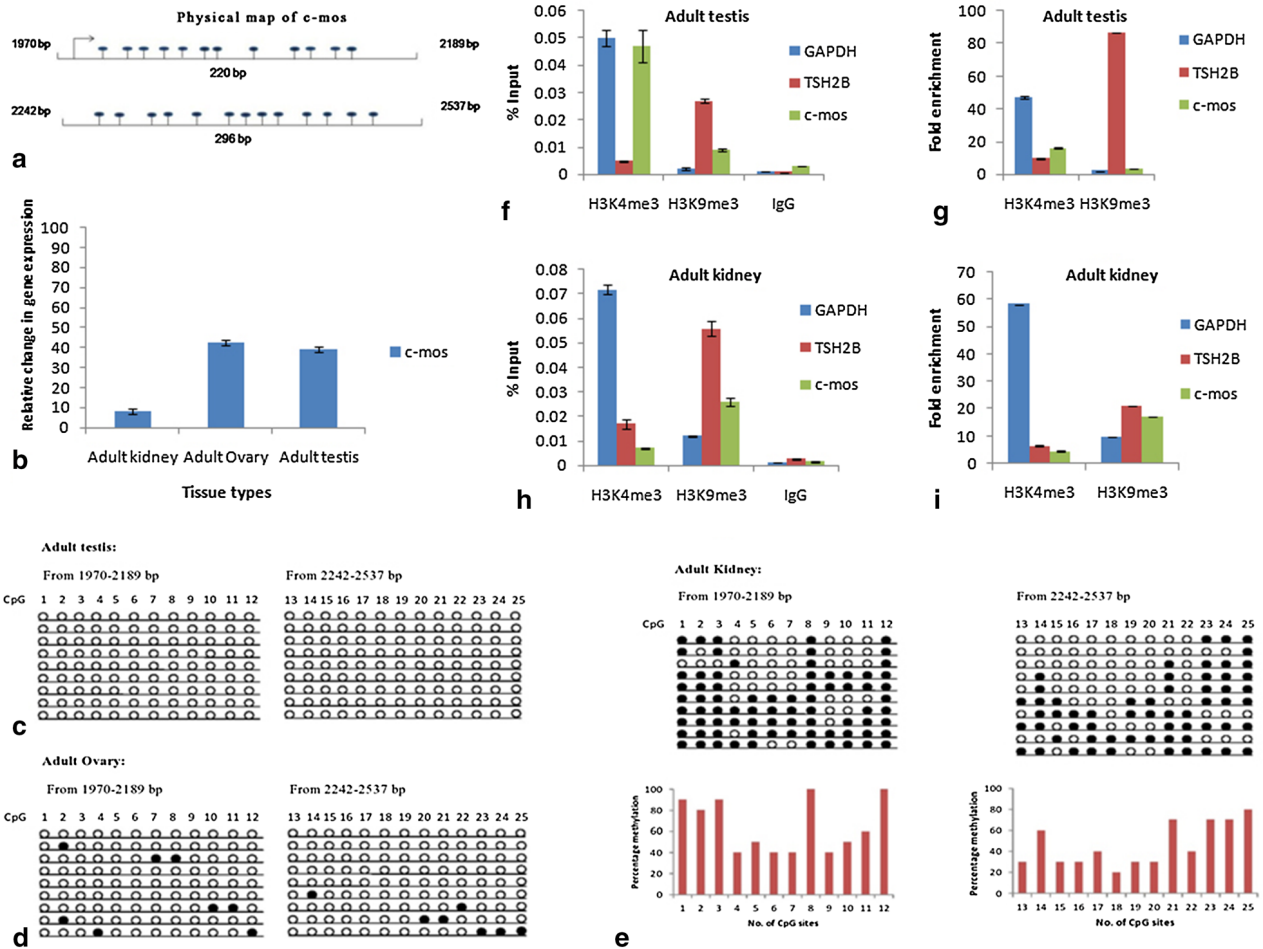
**a** Schematic diagram showing methylated CpG (*dark circles*) sites in the coding region of c-mos gene **b** Quantitative real-time PCR for analysis of relative gene expression in c-mos gene for different tissue types. Experiments were performed thrice independently. All results are shown as the mean ± S.D and are considered significant at P < 0.05. **c** *Line diagram* shows methylation pattern of total 25 CpG sites in the regulatory region of c-mos gene. Each *individual line* represents specific clone consisting of 25 CpG sites. Methylated CpGs are denoted by *dark circles* while non-methylated ones are denoted by *white circles* (*upper panel*). *Graph* represents percentage methylation in individual sites for adult testis, **d** adult ovary and **e** adult kidney respectively (*lower panel*) **f**–**i** *Graph* shows percentage input and fold enrichment done by ChIP-qPCR to assess the H3K4me3 and H3K9me3 occupancy of c-mos gene in adult testis and adult kidney. *Error bar* represents the mean ± SD

c-mos gene was analyzed for its DNA methylation patterns in both somatic and germinal tissues through sodium bisulfite genomic DNA sequencing with the aim at distinguishing methylated cytosines from unmethylated cytosine residues. Methylation was mapped in the regulatory/coding region of c-mos gene targeting a total of 25 CpG sites. Two pairs of primers covering 12 and 13 CpG sites respectively (from 1970–2189 and 2242–2537 bp) were designed with the PCR amplicon sizes of 220 and 296 bp respectively (Fig. 1a). In case of germinal tissues, adult testis shows no methylation as shown in (Fig. 1c) while adult ovary has a very few sites methylated (Fig. 1d). The methylation pattern in somatic tissues (adult kidney) is about 55% and seems to be heterogeneous and asynchronous (Fig. 1e) while germinal tissues possess only about 7% methylation. The methylation data for adult kidney shows that sites #8 and #12 were fully methylated while sites #1, #2, #3, #11, #14, #21, #23, #24 and #25 were more than 50% methylated and sites #5 and #10 showed 50% methylation. The remaining sites (#4, #6, #7, #9, #13, #15, #16, #17, #18, #19, #20 and #22) were less than 50% methylated. The real time quantitative gene expression data for c-mos showed an increased expression in adult testis and ovary whereas adult kidney showed repressed expression (Fig. 1b).

### ChIP (Chromatin-Immunoprecipitation) results

The results of Chromatin-Immunoprecipitation demonstrate the occupancy of the fractionated DNA fragments precipitated with a particular antibody against activated (H3K4me3) or repressed (H3K9me3) chromatin domains for specific gene in adult, fetal and neonatal stages of various somatic and germinal tissues of mice. The data were represented as percentage input and in terms of fold enrichment (FE).

#### c-mos

The chromatin interaction results of c-mos gene in adult testis illustrates that the percentage input of activated chromatin (H3K4me3) was higher than adult kidney (Fig. 1f, h). In a similar way fold enrichment was also higher (19 fold) in activated chromatin regions of adult testis as compared to adult kidney (Fig. 1g, i).

#### HoxB5

**Fig. 2:**
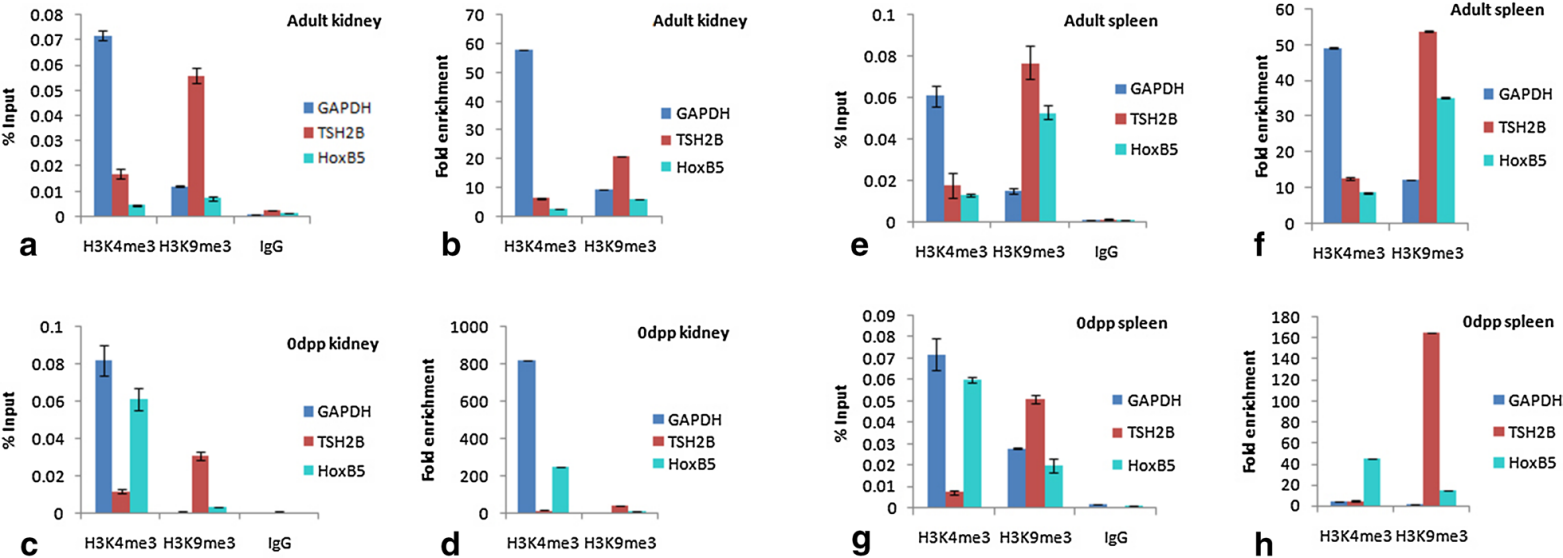
*Graphs* show percentage input and fold enrichment done by ChIP-qPCR to assess the H3K4me3 and H3K9me3 occupancy of HoxB5 gene in **a**, **b** adult kidney **c**, **d** 0dpp kidney **e**, **f** adult spleen **g**, **h** 0dpp spleen. *Error bar* represents the mean ± SD

The previous studies on HoxB5 promoter methylation by Sachan et al. (2006) demonstrated that fetal tissues of kidney and spleen showed expression of HoxB5 whereas no expression was detected in adult tissues of kidney and spleen. Higher methylation in adult kidney (18.6%) and adult spleen (74.55%) was observed in contrast to fetal kidney (2%) and fetal spleen (30.45%). We have performed ChIP for these tissues of adult and fetal stages. The ChIP results of HoxB5 gene in fetal kidney and spleen illustrate that the percentage input of activated chromatin (H3K4me3) was higher as compared to adult kidney and spleen (Fig. 2a, c, e, g). Fold enrichment data measured lower fold change in adult kidney when it is compared to fetal kidney. In contrast fetal kidney possesses 18 fold higher enrichment of activated chromatin in contrast to repressed (Fig. 2b, d). Similarly fold enrichment values showed higher fold change (3 times) for activated chromatin regions in the fetal spleen and (4 times) for repressed chromatin (H3K9me3) in adult spleen (Fig. 2f, h).

#### Sox11

**Fig. 3:**
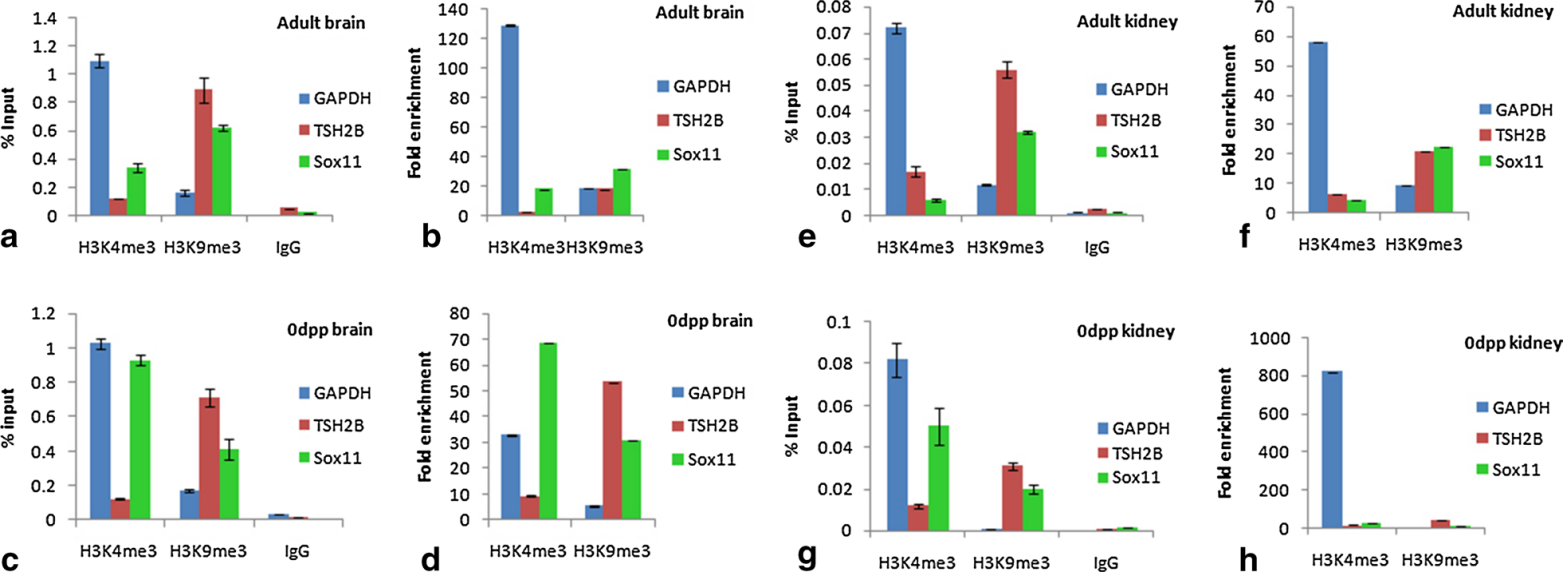
*Graphs* show percentage input and fold enrichment done by ChIP-qPCR to assess the H3K4me3 and H3K9me3 occupancy of Sox11 gene in **a**, **b** adult brain **c**, **d** 0dpp brain **e**, **f** adult kidney **g**, **h** 0dpp kidney. *Error bar* represents the mean ± SD

ChIP data for Sox11 shows that the higher percentage input of activated chromatin (H3K4me3) fraction was measured in the 0dpp brain as compared to adult brain and the same is true for 0dpp kidney and adult kidney respectively (Fig. 3a, c, e, g). Fold enrichment values were 2.2 fold higher in the 0dpp brain for activated chromatin regions, while adult brain has 1.7 times higher enrichment for repressed chromatin (Fig. 3d, b). Higher fold enrichment was found (2.0 fold) in activated chromatin regions for 0dpp kidney while adult kidney shows 5 times higher fold enrichment in repressed chromatin regions (Fig. 3h, f).

#### Sry

**Fig. 4:**
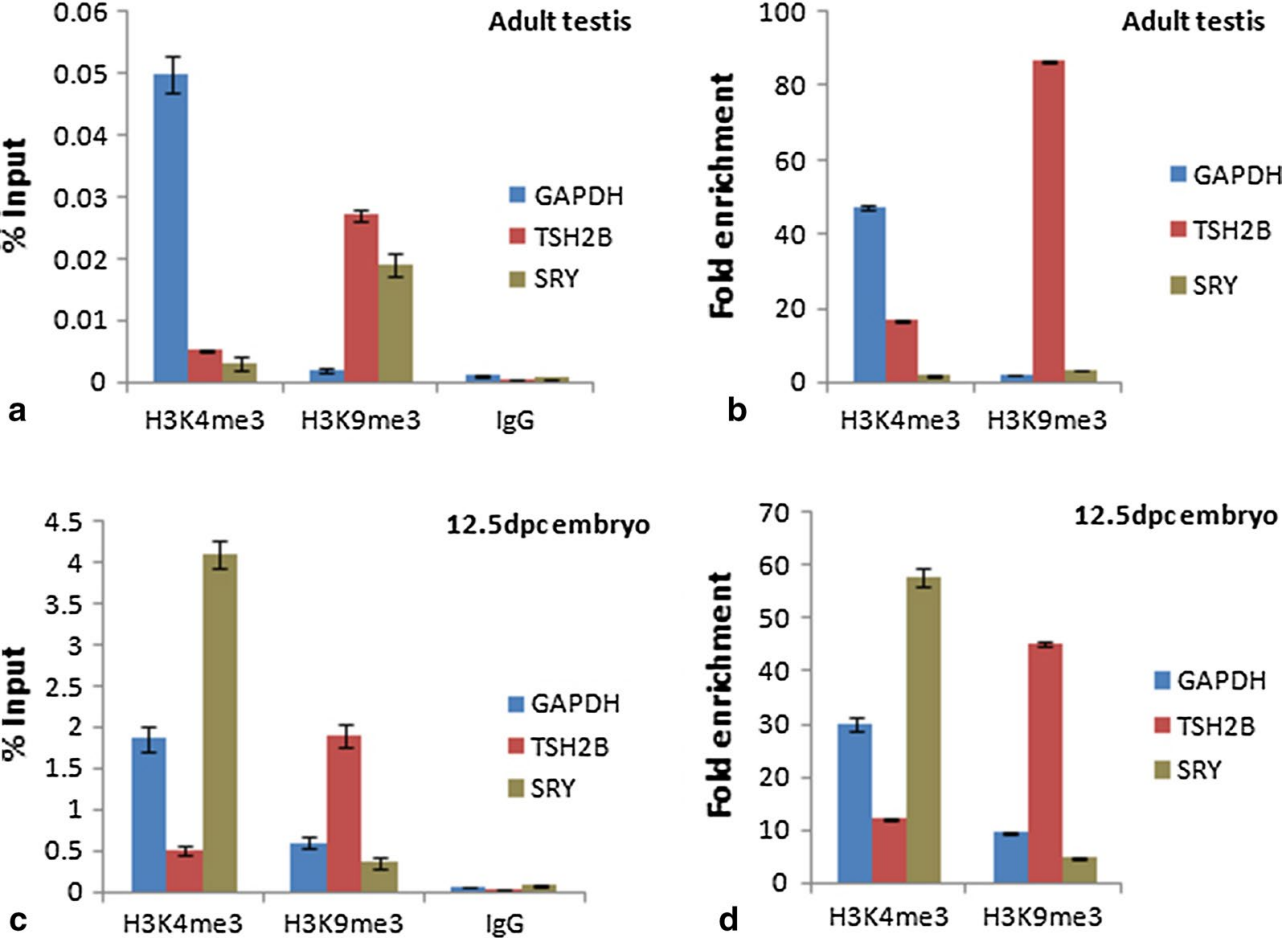
*Graph* show percentage input and fold enrichment done by ChIP-qPCR to assess the H3K4me3 and H3K9me3 occupancy of Sry gene in **a**, **b** adult testis **c**, **d** 12.5dpc embryo. *Error bar* represents the mean ± SD

The data suggest that the higher percentage input was observed in the activated chromatin region (H3K4me3) in 12.5dpc testis (MGCs) while higher repressed chromatin marks (H3K9me3) were found in adult testis (Fig. 4c, a). Fold enrichment was approximately 12 times higher for activated chromatin in 12.5dpc testis while sixfolds higher enrichment for the repressed region in adult testis (Fig. 4d, b).

## Discussion

The heterogeneous methylation pattern present in the somatic tissues (adult kidney shows the average percentage of all CpGs around 55) and almost complete absence of methylation in the germinal tissues (testis and ovary) of c-mos gene correlates inversely with its tissue specific expression. c-mos displays higher expression in adult testis and ovary and almost no expression in somatic tissues. It further strengthens the crucial role of c-mos gene in meiotic maturation of germinal tissues [14, 15] and in spermatogenesis [16] and oogenesis [17]. These finding correlates with the chromatin–immunoprecipitation results that displays the fractionation of chromatin fragments having the higher percentage input of activated chromatin regions (H3K4me3) in the expressing tissue (germinal tissues) in contrast to repressed chromatin regions (H3K9me3) in non-expressing tissues (somatic). Similarly HoxB5, a member of Hox family genes playing important roles in antero-posterior axial body patterning and the RNA transcripts were found in a wide range of fetal tissues such as lung, gut, kidney, liver and spleen [18–20]. Examination of the methylation pattern of HoxB5 shows higher methylation density in adult as compared to fetal stages of mouse tissues (Kidney and spleen) [11]. ChIP data also suggests higher input fraction of activated chromatin during fetal/neonatal stages of tissues in comparison to their adult counter parts.

Another developmental gene, Sox11, whose expression was prominent in developing central nervous system of mouse embryos, suggests its important role in neuronal growth, maturation and survival [21–23]. Promoter methylation mapping of Sox11 reveals higher methylation in both adult brain and kidney (average percentage of all CpGs around 48 and 68% in brain and kidney respectively) while the fetal and neonatal stages of the same tissues showed lower methylation density (14 and 31% in fetal brain and kidney respectively) [12]. The ChIP results of Sox11 in the present study are highly consistent with its tissue specific gene expression which correlates with changes in chromatin conformation.

A study by Nishino et al. [13] demonstrate that the mechanistic role of Sry gene regulation occurs by DNA methylation during mouse gonadal development and the Sry transcripts were expressed only in pre Sertoli cells of developing male gonad during 10.5–12.5dpc (average percentage of all CpGs around 62% and <3% in 11.5dpc liver and gonads respectively) [24, 25]. Hypermethylated status of the two regions analyzed was maintained in the tissues that did not express Sry however the methylation pattern was not purely consistent. Our ChIP results coincides with this finding as higher percentage input and fold enrichment values of activated chromatin fragments in fetal mesonephros gonads are in order with tissue specific gene expression [13] and inversely correlates with the promoter methylation of Sry gene.

Curradi et al. [26] analyzed the inhibitory effects of promoter DNA methylation through in vitro experiments and the results showed that a definite number of methylated CpG residues are required to organize a stable, diffusible chromatin structure. Histone deacetylation significantly imparts to transcriptional repression only when the number of modified cytosine residues is adequate to suppress the gene over a long range. These data suggest the importance of methylated cytosine residues for its ability to create a repressive effect and this repression can propagate in both directions for several hundred base pairs of the altered DNA only when enough number of methylated CpGs are present [26].

The potential role of DNA methylation is conserved from yeast to mammals. Some recent reports exhibited tissue and life-stage specific gene expression and its regulation by DNA methylation in species other than mammals suggesting functional similarity between transcriptional regulation [27, 28]. However, other studies also show impact of heterogeneous DNA methylation on gene expression including Oct-4 whose expression is confined to the germ line and pluripotent stem cells, which is critical for normal mouse development. The epigenetic regulation of Oct-4 show heterogeneous DNA methylation in its regulatory region among the population of adult somatic cells [29]. Similarly the heterogeneity in DNA methylation patterns across the distinct promoter and 5′UTR regions of differentiating stem cells and during reprogramming events suggest new insight into the methylation dynamics pertaining to embryonic stem cells [30, 31]. It has also been reported that the methylation patterns present in several imprinted genes and repetitive elements of embryonic germ (EG) cells may exhibit DNA methylation changes occurring in the germ cell lineage during the differentiation process [32]. Induction of mosaic DNA methylation patterns also leads to tumorigenesis by participating in important cancer related pathways and thus causing loss of co-expression connectivity in colorectal cancer [33]. The presence of epigenetic heterogeneity of developmentally important genes has important implications for assisted reproduction outcomes [34]. Another significant role was observed in the epigenetic regulation of stress reactivity in humans [35] and the formation and maintenance of memory in plasticity genes [36].

These findings elucidate that the tissue and stage specific gene regulation of several developmentally important genes of mouse is mediated through promoter/regulatory regions DNA methylation. The ChIP results are also consistently indicating a direct correlation between the percentage of input chromatin fraction and the level of gene expression.

## Conclusions

Transcriptional regulation mediated by the establishment of tissue specific methylation patterns in the promoter or regulatory regions of genes examined in this study might speculate to be a crucial mechanism which may be different from non-developmental genes. This might reflect the specialized functions of various developmental genes during the process of embryonic development. A significant level of heterogeneous/asynchronous methylation exists as the development progresses from embryo to adult and in most of the cases this heterogeneity is associated with transcriptional gene regulation and chromatin modifications. Further, higher density of methylation present in the promoter regions might render the DNA inaccessible (either fully or partially) for binding of transcription factors, leading to altered chromatin assembly and gene expression.

## Abbreviations

dpc: days post coitum
dpp: days post partum
ChIP: chromatin immuno-precipitation
MeCP2: methyl cytosine binding protein 2
